# Polygenic Hazard Score Associated Multimodal Brain Networks Along the Alzheimer’s Disease Continuum

**DOI:** 10.3389/fnagi.2021.725246

**Published:** 2021-09-03

**Authors:** Kaicheng Li, Zening Fu, Shile Qi, Xiao Luo, Qingze Zeng, Xiaopei Xu, Peiyu Huang, Minming Zhang, Vince D. Calhoun

**Affiliations:** ^1^Tri-Institutional Center for Translational Research in Neuroimaging and Data Science (TReNDS), Georgia Institute of Technology, Georgia State University, Emory University, Atlanta, GA, United States; ^2^Department of Radiology, The Second Affiliated Hospital of Zhejiang University School of Medicine, Hangzhou, China; ^3^Department of Computer Science and Engineering, Nanjing University of Aeronautics and Astronautics, Nanjing, China; ^4^Department of Psychology, Computer Science, Neuroscience Institute, and Physics, Georgia State University, Atlanta, GA, United States; ^5^Department of Electrical and Computer Engineering, Georgia Institute of Technology, Atlanta, GA, United States

**Keywords:** Alzheimer’s disease, polygenic hazard score, supervised multimodal fusion, default mode network, executive control network, visuospatial network, cognitive decline

## Abstract

**Background:**

Late-onset Alzheimer’s disease (AD) is a polygenic neurodegenerative disease. Identifying the neuroimaging phenotypes behind the genetic predisposition of AD is critical to the understanding of AD pathogenesis. Two major questions which previous studies have led to are: (1) should the general “polygenic hazard score” (PHS) be a good choice to identify the individual genetic risk for AD; and (2) should researchers also include inter-modality relationships in the analyses considering these may provide complementary information about the AD etiology.

**Methods:**

We collected 88 healthy controls, 77 patients with mild cognitive impairment (MCI), and 22 AD patients to simulate the AD continuum included from the ADNI database. PHS-guided multimodal fusion was used to investigate the impact of PHS on multimodal brain networks in AD-continuum by maximizing both inter-modality association and reference-modality correlation. Fractional amplitude of low frequency fluctuations, gray matter (GM) volume, and amyloid standard uptake value ratios were included as neuroimaging features. Eventually, the changes in neuroimaging features along AD continuum were investigated, and relationships between cognitive performance and identified PHS associated multimodal components were established.

**Results:**

We found that PHS was associated with multimodal brain networks, which showed different functional and structural impairments under increased amyloid deposits. Notably, along with AD progression, functional impairment occurred before GM atrophy, amyloid deposition started from the MCI stage and progressively increased throughout the disease continuum.

**Conclusion:**

PHS is associated with multi-facets of brain impairments along the AD continuum, including cognitive dysfunction, pathological deposition, which might underpin the AD pathogenesis.

## Introduction

Late-onset Alzheimer’s disease (LOAD) is the most common form of dementia, whose morbidity and progression is largely associated with the risk genes (Escott-Price and Jones, 2017). Genome-wide associated studies (GWAS) have identified multiple AD risk genes, including apolipoprotein E (APOE) ε4, CLU, BIN1, PICALM, MS4A, ABCA7, and CR1 (Lambert et al., 2013; Desikan et al., 2015). These genetic variants were involved in the downstream molecular pathways and affect the AD pathological substances, like amyloid production and clearance, highlighting the importance of genetic variants in AD pathogenesis.

Identifying neuroimaging phenotypes of AD genetic risk may be critical to the understanding of AD pathogenesis (Biffi et al., 2010; Cruchaga et al., 2013). For instance, the APOE ε4 allele, as the strongest AD risk gene (Yu et al., 2014), was found to be associated with brain atrophy and amyloid deposition involving the hippocampus and temporoparietal regions (Li et al., 2017), as well as decreased functional connectivity in the default mode network (DMN) (Machulda et al., 2011). Besides, the BIN1 gene, which is the second crucial genetic susceptibility locus for LOAD, was associated with the rate of volume change in the left parahippocampal and right inferior parietal (Li et al., 2017). Moreover, other genetic variants like the CR1 (Crehan et al., 2012) and SORL1 (Scherzer et al., 2004) were also found to be involved in brain clearance thus were regarded as the crucial components in the pathogenesis of AD. To further elaborate, SORL1 showed association with hippocampal atrophy (Cuenco et al., 2008) and functional connectivity impairments (Shen et al., 2017). Notably, another APOE major genetic variant, namely APOE ε2 allele is related to a reduced risk of AD thus seems to confer a protective effect against AD (Reiman et al., 2020). These findings provided some hints on how genetic variants affect the occurrence of AD acting through pathological, structural, and functional brain alterations. However, there are still two major problems remain unsolved about the relationship between neuroimaging and gene in AD.

On the one hand, LOAD is a polygenic disorder. Any single genetic variant found by previous studies cannot fully reflect the total genetic risk of AD (Escott-Price and Jones, 2017). A complex genetic index combining all risk and potentially protective genetic variants should be a better choice to identify an individual’s overall genetic risk for AD (Escott-Price et al., 2015). Recently, the polygenic hazard score (PHS), which is calculated by integrating multiple genetic variants (APOE and 31 other genetic variants) (Desikan et al., 2017), is suggested to be a better index for evaluating the global impact of AD susceptibility variants (Mormino et al., 2016; Ge et al., 2018). Clinically, PHS is effective in predicting individual onset age of AD dementia, even among APOE ε3/3 individuals, who constitute the majority of all individuals with AD (Desikan et al., 2017). Furthermore, PHS shows a significant correlation with longitudinal cognitive decline in AD (Tan et al., 2017; Kauppi et al., 2018). The possible mechanism may be the effect of PHS on the structural and functional brain alterations as well as AD pathological deposition. Accordingly, further neuroimaging research should be performed to explore the effect of PHS on the brain.

On the other hand, most previous studies are based on the single-modality analysis, neglecting the potential inter-modality relationships. Some studies observed the spatial overlaps between different neuroimaging features in AD. For example, the spatial distribution of Aβ (Buckner et al., 2005; Mormino et al., 2011; Kvavilashvili et al., 2020) and Tau (Buckner et al., 2008) is highly overlapped with functional and structural impairments in DMN regions in AD. Such spatial similarity indicates the inter-modal information association, which may provide complementary information about the clues of AD etiology. Therefore, multimodal fusion is an effective analysis strategy that could jointly investigate multimodal data and detect their co-alterations related to diseases (Li et al., 2009; Sui et al., 2012). A recent approach for reference guided multimodal fusion method, called multisite canonical correlation analysis with reference + joint independent component analysis (MCCAR + jICA) (Qi et al., 2018a), shows high effectiveness in exploring components of interest related to a particular trait, for example, gene and cognitive score. MCCAR + jICA uses subject-wise clinical measures as the reference to guide a 3-way MRI fusion and by maximizing both inter-modality association and reference-modality correlation. For example, Qi et al. (2018b). used the gene as the reference and successfully identified the risk gene-associated patterns in major depressive disorder. Accordingly, the appliance of the MCCAR + jICA model with PHS as reference is an ideal method for comprehensively evaluating the neuroimaging phenotypes of PHS in AD-continuum subjects.

Accordingly, the current study aimed to explore the PHS associated multimodal brain alterations in AD using MCCAR + jICA. We included healthy control (HC), mild cognitive impairments (MCI), and AD subjects to simulate the AD continuum and observed how the identified multimodal pattern alternates during AD evolution. Using subject-wise PHS as the reference, three features from different magnetic resonance imaging (MRI) modalities [fractional amplitude of low frequency fluctuations (fALFF) from resting-state functional MRI (rs-fMRI), GMV from structural MRI, and voxel-wise amyloid standard uptake value ratios (SUVR) from^18^F-florbetapir PET ([^18^F]-AV45 PET)] were analyzed jointly to leverage the cross-information in the existing data that cannot be detected by single modality analysis alone. Based on the previous findings, we propose that (1) the PHS associated multimodal brain alterations mainly involve core brain networks, like DMN; and (2) such degenerative pattern evolves with the progression of AD. Thereinto, functional and amyloid changes may occur earlier while GM atrophy takes place later.

## Materials and Methods

### Study Participants

Data used in the current study were obtained from the Alzheimer’s disease neuroimaging initiative (ADNI) database (Supplementary Material 1 provides detailed information about ADNI). All included subjects underwent the T1-weighted structural scan, [^18^F]-AV45 PET, rsfMRI, PHS, and comprehensive neuropsychological assessments (Supplementary Material 2 provides detailed information about the MRI and PET acquisition). All the cognitive status and MRI data were obtained from the same visit. This criterion yielded 88 HC, 77 MCI, and 22 AD patients from the ADNI database (see flowchart and inclusion criteria in Supplementary Material 3).

### Neuropsychological Assessment

All subjects completed comprehensive neuropsychological tests, including assessment of general mental status (MMSE and CDR), memory [ADNI memory composite score (ADNI-MEM)], executive function [ADNI executive function composite score (ADNI-EF)], language function [ADNI language function composite score (ADNI-LAN)], and visuospatial function [ADNI visuospatial function composite score (ADNI-VS)]. More detailed information about composite cognitive scores is provided in Supplementary Material 4.

### Polygenic Hazard Score (PHS)

PHS was developed and validated by Desikan et al. (2017). Firstly, common variants associated with AD (at *p* < 10^–5^) were identified from 17,008 AD cases and 37,154 controls from Stage 1 of the International genetics of Alzheimer’s Project. Then, based on the Alzheimer’s Disease Genetic consortia phase 1 genetic data [excluding individuals from the ADNI and National Institute of Aging Alzheimer’s Disease Center (NIA ADC) samples], a stepwise Cox proportional hazard model was applied to examine the association between these SNPs and AD while controlling for the effects of gender, APOE variants, and the top five genetic principal components (to control for the effects of population stratification). This model identified 31 SNPs, including the APOE ε2 and APOE ε4 genotype, which were further used to derive the individual PHS. Finally, the PHS was integrated with population-based incidence rates from the US population to provide estimates of instantaneous risk for developing AD. The PHS represents the vector product of an individual’s genotype for the 31 SNPs and the corresponding parameter estimates from the Cox proportional hazard model.

Detailed information on the PHS calculation can be found on the ADNI website^1^. PHS data used in the current study are publicly available from the ADNI database^2^.

### Image Preprocessing

RsfMRI data preprocessing was performed using the data processing assistant and resting-state fMRI toolbox (DPARSF)^3^ (Chao-Gan and Yu-Feng, 2010) based on Statistical Parametric Mapping 12 (SPM12)^4^. The first 5 rsfMRI scans were discarded for the signal equilibrium and subject’s adaptation to the scanning noise (Chao-Gan and Yu-Feng, 2010). The remaining 135 images were corrected for timing differences in slice acquisition. After that, a rigid body motion correction was performed to correct the head motion of the fMRI scans. Then, the mean rsfMRI image was co-registered to the subject-specific T1 image and spatially normalized to the Montreal Neurological Institute (MNI) standard space, resampling into 3 × 3 × 3 mm^3^ cubic voxel. Scrubbing was then performed to reduce motion-related artifacts by using a framewise displacement threshold of 0.5 (Power et al., 2012). To control the residual effects of motion and other non-neuronal factors, we removed covariates, including six head motion parameters and signals of white matter (WM) and cerebrospinal fluid (CSF) (Friston et al., 1996; Chao-Gan and Yu-Feng, 2010). Finally, the fMRI data were smoothed using an 8 mm full width at half maximum kernel (FWHM).

The T1-weighted image preprocessing was performed using voxel-based morphometry analysis based on SPM12. Briefly, T1-weighted scans were aligned to the T1-weighted template image. Secondly, the aligned images were segmented into GM, WM, and CSF compartments with bias correction. Then, the GM maps were normalized to MNI coordinate space via the modulated method, resampling to 3 × 3 × 3 mm^3^ voxel size. Finally, GMV was calculated by the modulated method was smoothed using an 8 mm FWHM Gaussian kernel.

The [^18^F]-AV45 PET preprocessing was performed using the PET-PVE12 (an SPM toolbox for Partial Volume Effects (PVE) correction in brain PET (Gonzalez-Escamilla et al., 2017). Briefly, the T1-weighted image was firstly segmented into different tissue compartments (GM, WM, and CSF) based on an adaptive maximum a posterior approach with partial volume estimation. An iterative hidden Markov random field model (Cuadra et al., 2005) was further applied to remove isolated/unclassified voxels. Then, [^18^F]-AV45 PET data were co-registered to the structural MRI data and corrected for PVE using the voxel-wise method defined by Muller-Gartner et al. (1992) (PVEc-MG) methods. Here, we set the isotropic point spread function at 8 mm according to the effective image resolution of the ADNI AV45 PET data. Then, the voxel-wise [^18^F]-AV45 PET SUVR map was calculated using the whole cerebellar signal in the individual raw PET images as the reference. Finally, for voxel-based analyses, PVEc-MG corrected [^18^F]-AV45 PET images were spatially warped using the deformation fields derived from registration of the co-registered MRI scans to the reference template. Finally, warped images were smoothed with an 8 mm FWHM Gaussian kernel.

### Feature Extraction

Three representative neuroimaging features (fALFF, GMV, and [^18^F]-AV45 PET SUVR) were calculated as the input of fusion analysis. Voxel-wise GMV and [^18^F]-AV45 PET SUVR map (amyloid SUVR) were directly obtained after the preprocessing. Notably, we considered atrophy, reflected by volume change, as the signature of GM atrophy. The fALFF is the ratio of power spectrum of low-frequency to that of the entire frequency range (Zou et al., 2008) which was calculated using the DPARSF toolbox. The time series of voxels were first converted into the frequency domain using a fast Fourier transform. We computed the square root of the power spectrum. The averaged square root was obtained across 0.01–0.1 Hz and across the whole frequency band. Then we calculated the ratio of averaged square root in low frequency band to that in the entire frequency band as the fALFF for each voxel. Finally, normalization is done separately for each feature within groups, using the square root of mean of squared data for all subjects.

### Fusion With Reference

**FIGURE 1:**
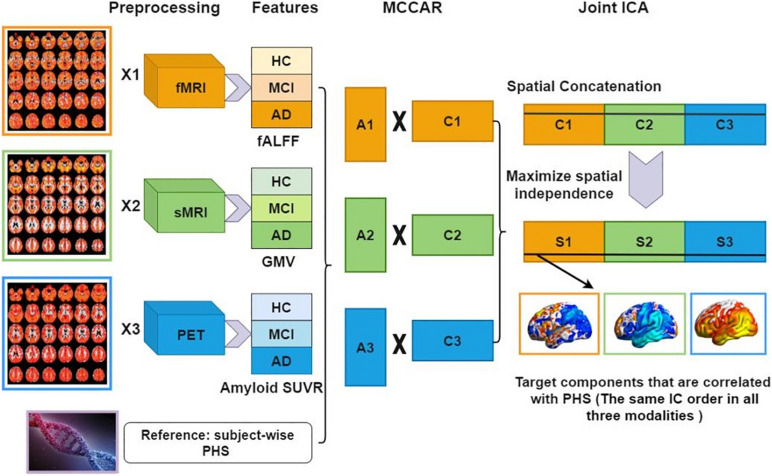
Data analysis flowchart using fusion with reference. Extracted neuroimaging features (fALFF, GMV, and amyloid SUVR) are first stacked to 2D matrices for each modality. Then, PHS is used as the reference to guide the joint decomposition of three features to generate spatial maps **(C1,C2,C3)** and their corresponding canonical variants **(A1,A2,A3)**. Finally, joint ICA is applied on the concatenated spatial maps to obtain the final ICs and their corresponding mixing matrices. fMRI, functional MRI; sMRI, structural MRI; HC, healthy control; MCI, mild cognitive impairment; AD, Alzheimer’s disease; fALFF, fractional amplitude of low frequency fluctuations; GMV, gray matter volume; SUVR, standard uptake value ratios; PHS, polygenic hazard score; MCCAR, multisite canonical correlation analysis with reference; ICA, independent component analysis; IC, independent components.

The normalized features were jointly analyzed based on MCCAR + jICA (Qi et al., 2018a) using the Fusion ICA Toolbox (FIT).^5^ Figure 1 shows a detailed analysis flowchart. Firstly, for each modality, the neuroimaging features were stacked to 2D matrices with the row indicates the subject and the column indicates the features. Then, PHS was used as the reference to guide the joint decomposition of three features to generate spatial maps and their corresponding canonical variants for each modality. MCCAR identifies joint multimodal components that show maximal correlation with the reference and inter-modality covariation based on supervised learning. Based on the modified minimum description length criterion (Li et al., 2007), 15 components were estimated for each feature (fALFF, GMV, and amyloid SUVR). Finally, jICA is applied to the concatenated spatial maps to obtain the final independent components (ICs) and their corresponding mixing matrices. More details of the model are shown in Qi et al. (2018a).

Analysis of variance (ANOVA) was performed to explore the difference of mixing coefficients of each component for each modality. Then, *post hoc* analysis using a two-sample *t*-test was performed to examine the source of ANOVA difference (significant at *p* < 0.05, false discovery rate (FDR) corrected).

### Correlation Between Features and Cognitive Scores

To explore whether the multimodal brain alterations underpin the cognitive decline, we further examined the potential relationship between mixing coefficients of multimodal components and cognitive performance (i.e., memory, executive, language, and visuospatial function). The Pearson correlation between the loadings of features and cognitive scores (ADNI-MEM, ADNI-EF, ADNI-LAN, ADNI-VS) was calculated across subjects (significant at *p* < 0.05, FDR corrected).

We also performed the correlation analysis within every group (HC, MCI, and AD) to show the distinct association with cognition. To remove the possible effect of covariates (age, gender, and education level), we further performed partial correlation analysis. Detailed information was listed in Supplementary Material 6.

## Results

### Demographic and Neuropsychological Data

Detailed demographics are provided in Table 1. We used a Chi-squared test for categorical (gender, APOE genotype) and ANOVA for continuous data (age, education), respectively, (SPSS, version 19.0). Then, a two-sample *t*-test was performed to reveal the source of ANOVA difference (significant at *p* < 0.05).

**TABLE 1:** Demographic information.

Demographic characteristics	HC	MCI	AD	*F*-value/X^2^	Sig
	*N* = 88	*N* = 77	*N* = 22		
Age	77.40 ± 6.13	77.13 ± 8.14	80.70 ± 7.83	2.21	0.11
Gender (F/M)	42/46	32/45	9/13	0.76	0.69
Education	16.42 ± 2.68	15.94 ± 2.95	15.50 ± 2.86	1.20	0.30
APOE 4	3/88	30/77	12/22	41.08	<0.001^ab^
GDS	0.88 ± 1.11	1.43 ± 1.20	2.36 ± 1.65	14.11	<0.001^abc^
PHS	−0.29 ± 0.29	0.26 ± 0.82	0.46 ± 0.62	23.91	<0.001^ab^
**Cognitive scores**					
MMSE	29.05 ± 1.10	28.22 ± 1.67	19.41 ± 5.22	174.95	<0.001^abc^
CDR global	0.00 ± 0.00	0.50 ± 0.00	1.11 ± 0.55	360.12	<0.001^abc^
CDR sum	0.05 ± 0.15	1.48 ± 0.96	6.27 ± 3.05	235.63	<0.001^abc^
ADNI_MEM	1.10 ± 0.63(87/88)	0.34 ± 0.53(77/77)	−0.88 ± 0.70(22/22)	103.78	<0.001^abc^
ADNI_EF	1.05 ± 0.81(87/88)	0.49 ± 0.88(76/77)	−0.94 ± 0.99(20/22)	44.63	<0.001^abc^
ADNI_LAN	0.95 ± 0.65(88/88)	0.42 ± 0.80(77/77)	−0.89 ± 1.23(22/22)	47.16	<0.001^abc^
ADNI_VS	0.18 ± 0.66(88/88)	−0.02 ± 0.78(77/77)	−0.88 ± 1.13(22/22)	16.33	<0.001^bc^

*HC, healthy control; MCI, mild cognitive impairment; AD, Alzheimer’s disease; APOE, apolipoprotein; GDS, geriatric depression scale; PHS, polygenic hazard score; MMSE, Mini-Mental State Examination; CDR, clinical dementia rating; ADNI-MEM, the composite scores for memory in ADNI; ADNI-EF, the composite scores for executive function in ADNI; ADNI-LAN, the composite scores for language in ADNI; ADNI-VS, the composite scores for visuospatial function in ADNI. a–c, post hoc analysis further revealed the source of ANOVA difference (^*a*^HC vs. MCI; ^*b*^HC vs. AD; ^*c*^MCI vs. AD) (p < 0.05, significant difference between groups). Data are presented as means ± standard deviations. There are some missing values in multiple composite cognitive scores. We thus listed the proportions. Notably, no missing values in other demographic information.*

There is no group difference in age, gender, and education among HC, MCI, and AD. MCI and AD had higher PHS, APOE 4 percentage, and GDS compared to HC. In terms of the cognitive level, MCI and AD had lower cognitive scores in all items compared to HC.

### PHS Associated Multimodal Covarying Imaging Patterns

**FIGURE 2:**
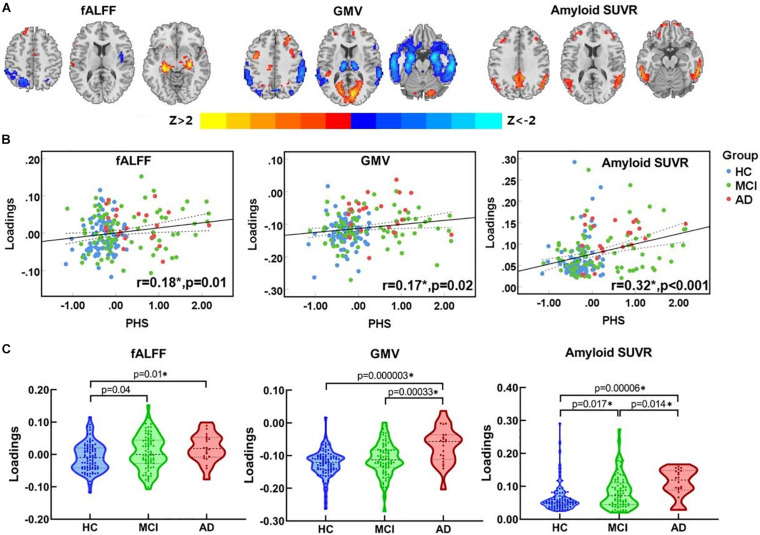
The identified joint component. **(A)** The spatial maps visualized at |Z| > 2, where the positive *Z*-values (red regions) means higher fALFF, GMV, and more amyloid deposition, and negative *Z*-values (blue regions) indicate decreased fALFF, GMV, and less amyloid deposition. **(B)** The loadings of the identified joint component and PHS were positively correlated (HC, blue dots; MCI, purple square; AD, red triangle) **(C)** Boxplot of the loading parameters of the identified joint component. *Significant at *p* < 0.05, FDR corrected. fALFF, fractional amplitude of low frequency fluctuations; GM, gray matter; SUVR, standard uptake value ratios; PHS, polygenic hazard score; HC, healthy control; MCI, mild cognitive decline; AD, Alzheimer’s disease.

One joint component was identified that was correlated with PHS and showed significant alteration along the AD continuum (HC, MCI, AD). The resulting spatial maps were Z-transformed and visualized at |Z| > 2 in Figure 2A. Along AD-continuum, PHS was correlated with (1) decreased fALFF in the precuneus, inferior parietal lobule (IPL) and middle temporal gyrus (MTG); (2) decreased GMV in the precuneus, IPL, and temporal region; and (3) increased amyloid SUVR in the precuneus, IPL, posterior cingulate cortex (PCC), and temporal regions. These commonly affected brain regions are essential components of the default mode network (DMN). The PHS was also associated with other covarying patterns along the AD continuum (HC, MCI, AD) such as (1) increased fALFF in the hippocampus, parahippocampal gyrus, and frontal regions; (2) increased GMV in the frontal and occipital regions; and (3) increased amyloid SUVR in the frontal regions. These involved frontal and occipital regions indicate the alterations of the executive control network (ECN) and visuospatial network, respectively. Detailed anatomical information of the identified regions in the joint component was summarized in Supplementary Material 5.

As shown in Figure 2B, the loadings of ICs were positively correlated with PHS (*r* = 0.18, *p* = 0.01 for fALFF; *r* = 0.17, *p* = 0.02 for GMV; *r* = 0.32, *p* < 0.001 for amyloid SUVR; *p*-values are FDR corrected). Significant differences in loadings of fALFF, GMV, and amyloid SUVR (Figure 2C) among groups were also observed. To be specific, both MCI and AD showed higher loadings than HC in fALFF. As for GMV, AD showed higher loadings than HC and MCI. Moreover, both MCI and AD showed higher loadings when compared to HC in amyloid SUVR; notably, AD showed higher loadings than MCI. The overall results indicate that the functional abnormalities occur during the early AD stage (HC to MCI), while the GM abnormalities occur during the late AD stage (MCI to AD). Continuous amyloid SUVR changes can be observed along the whole AD continuum (HC, MCI, and AD).

### Multimodal Features Associated With Cognition

**FIGURE 3:**
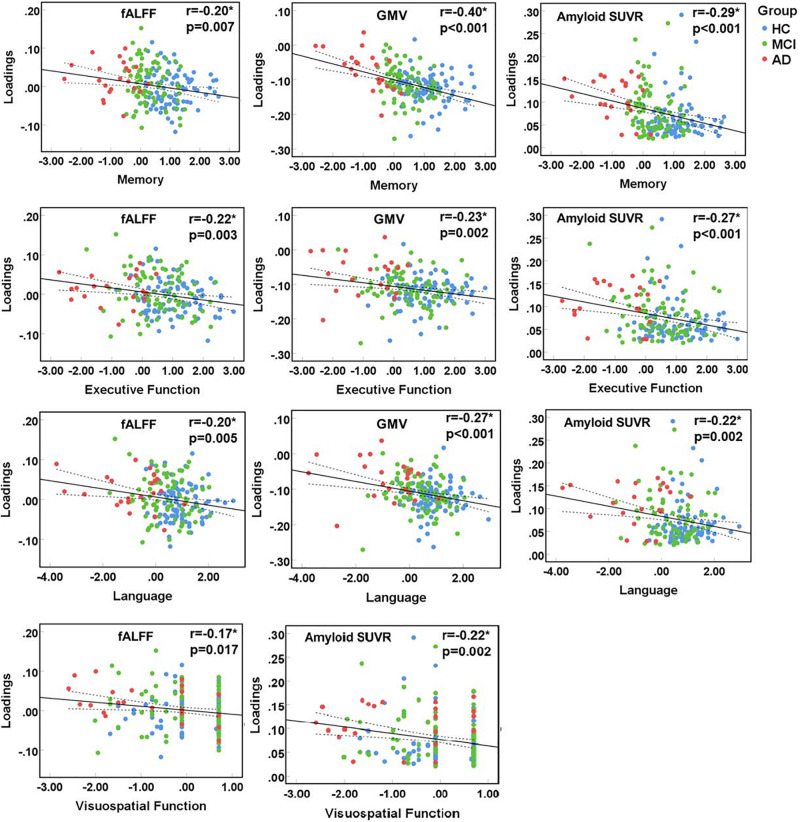
Correlation between loadings of the identified joint component and cognitive function. *Significant at *p* < 0.05, FDR corrected. HC, healthy control; MCI, mild cognitive impairment; AD, Alzheimer’s disease; fALFF, fractional amplitude of low frequency fluctuations; GMV, gray matter volume; SUVR, standard uptake value ratios.

The identified multimodal brain alterations were significantly associated with four major cognitive domains. The loadings of three features (fALFF, GMV, amyloid SUVR) were negatively correlated with memory, executive, language, and visuospatial function. Detailed results were listed in Figure 3 (significant at *p* < 0.05, FDR corrected) and Supplementary Material 6.

Furthermore, we tested the robustness of the current results from the following three aspects: (1) To further reduce the possible effect of covariates, we performed analysis corrections for age, gender as well as education and added them in the Supplementary Material 7. (2) To further clarify the effect of APOE and other SNPs, we supplementarily identified the role of APOE alone and the other SNPs on the brain changes from three aspects and added them in the Supplementary Material 8. (3) We repeated the fusion analysis in the HC with negative amyloid, MCI, and AD with positive amyloid and listed the results in the Supplementary Material 9.

## Discussion

Based on cross-sectional datasets spanning the continuum of AD, we explored the progressive pattern of PHS-associated multimodal impairments. We applied the PHS-guided multimodal fusion and identified the PHS-related multimodal covaried pattern, including DMN, ECN, visuospatial networks in function, pathological deposition, and GM atrophy that are correlated with multiple cognitive domains. Furthermore, along the AD continuum, amyloid deposition and functional impairment occurred earlier, followed by GM atrophy. Notably, amyloid deposition started from the early stage and progressively changed along with the disease. Collectively, the current study provided insight into the linkage between AD risk genes and multimodal neuroimaging covariations, which might underpin the pathophysiology of cognitive impairments in AD.

### PHS Associated Multimodal Pattern Involves Multiple Networks

Multimodal brain alterations in fALFF, GMV, and amyloid SUVR were identified to be related to PHS, which indicates that subject-specific PHS was associated with functional impairments, GM atrophy, and amyloid accumulation in AD susceptible brain region. Such multimodal alterations support previous findings, indicating amyloid as the key underlying mechanism that initiates AD onset and leads to downstream impairments, including brain dysfunction and neuron death in different brain regions.

These identified brain regions are spatially located within three networks including DMN, ECN, and visuospatial network, which played distinct roles in the onset and progression of AD. PHS related impairments converge on brain regions such as the precuneus, IPL, and temporal gyrus, which are the core components of DMN (Andrews-Hanna et al., 2014). Specifically, decreased functional connectivity, GM atrophy, and increased amyloid deposit manifest as decreased fALFF, GMV, and increased amyloid SUVR in the AD continuum (HC, MCI, AD) jointly suggested PHS-related multimodal impairments within DMN. These findings are consistent with but extend previous studies that the DMN is the most vulnerable region under AD attacks thus suffers severe functional and structural impairments (Buckner et al., 2008; Palmqvist et al., 2017). The association between increased amyloid deposit and decreased GMV in DMN supports the mechanism of amyloid burden associated structural impairments in AD.

The identified component also involves frontal regions, which spatially are the core components of the ECN (Weiler et al., 2014). Increased values in PHS associated fALFF, GMV, and amyloid SUVR suggest an increase in the volume and function of frontal regions, even under more amyloid pathological deposits. These findings are in line with the compensatory enlargement of synaptic size in the frontal cortex under AD pathology (Scheff et al., 1990), and indicate the increased volume, and functional connectivity in frontal regions may work as a compensatory function in the face of AD pathology (Becker et al., 2013). Interestingly, the joint increase in the frontal and hippocampus was only observed in the fMRI modality. This pattern partially proves the role of hippocampal-prefrontal interactions in cognitive disease and can be interpreted as a functional compensatory mechanism (Filbey et al., 2010).

We also observed PHS-related GMV increase in occipital regions, where the processing core of the visuospatial network is located. Notably, alterations in functional and amyloid features were not detected in occipital regions, suggesting that PHS-related visuospatial network impairments are mainly confined to GM atrophy. Similar conclusions can be found in previous studies which observed the retained physiological glucose metabolism in the occipital cortex in AD (Rice and Bisdas, 2017).

### Progressive Changes of Multimodal Patterns Suggest the AD Genetic Pathogenesis

Group comparisons showed a progressive increase in all PHS-related neuroimaging features with the development of AD, suggesting severer brain structural and functional impairments, as well as more pathological deposits, resulted from AD genetic risk. Similar conclusions can be made from previous studies using single modal analysis. For example, our previous study observed the progressive GMV loss along the AD continuum involving DMN and ECN (Li et al., 2019). Moreover, plenty of functional MRI studies showed progressive multiple network impairments in AD (Zeng et al., 2019). All the above evidence indicates that PHS affects multi-facets of the brain, and such genetic effect deepens with the development of AD.

Three features showed different patterns of change in different disease stages, which further expands our understanding of AD pathophysiology. MCI showed increased loadings of fALFF and amyloid SUVR when compared to HC while no significant changes in that of GMV, suggesting that the effects of AD genetic risk initially appear in functional MRI and pathological deposit. As the disease progresses, loadings of amyloid SUVR and GMV in AD showed a significant increase when compared to MCI, suggesting that GM atrophy occurs at a relatively late AD stage. Notably, the GMV and amyloid SUVR showed partial spatial overlap. This finding is comprehensible considering the late AD stage has higher possibilities of carrying both amyloid and Tau, which result in the GM atrophy. Moreover, the loading of amyloid SUVR showed a progressive increase throughout AD, indicating that amyloid deposition is the key mechanism underlying the multimodal neuroimaging changes. This is reasonable since the amyloid cascade hypothesis of AD postulates that the accumulation of amyloid occurs even decades before the onset of the clinical symptoms.

Such a time inconsistency of brain impairments supports the followed inference: functional alternations tend to happen at the preclinical stage of AD, while GM atrophy takes place at a relatively late AD stage. Thereinto, amyloid deposition may be the key to revealing the underlying mechanism.

### Multimodal Neuroimaging Features Correlate With Multiple Cognitive Scores in AD-Continuum Subjects

Cognition is formed based on the cooperation of multiple networks. Our study supported this theory by finding the significant correlations between the loadings of three features and multiple cognitive scores, including memory, executive, language, and visuospatial function. This is also supported by previous studies, which proposed brain network impairments as the underlying neural mechanism of multi-domain cognitive impairments in AD, involving the DMN, ECN, as well as other networks.

Notably, the identified neuroimaging features showed different associations to different cognitive domains. Our correlation analysis on the patient groups (MCI and AD) found the most significant association in memory. This is in line with the clinical symptoms, regarding memory loss as the earliest suffered and most significantly impaired cognitive function in AD. The underlying mechanism is the widespread functional and structural impairments in DMN, which is also proved in our current study. Notably, the most significant association was found between memory and GMV, which also makes sense since neuron is the basic unit for cognitive function. Visuospatial function is less associated with the multimodal changes in our study. This is consistent with clinical findings showing the relatively spared visuospatial function in AD. Our findings showing limited involvement in just GMV, with functional and pathological impairment spared, may help explain that. Similarly, one previous study also found that the amyloid deposit is much less associated with visual memory (Rice and Bisdas, 2017).

## Limitation

There are several limitations in the current study. First, the sample sizes of different groups are unbalanced, with a relatively small sample size for the AD group. Thus, the corresponding conclusion about AD should be treated cautiously. We would like to validate our findings in future studies with balanced subjects for each group. Secondly, the intrinsic causality between amyloid and fALFF, as well as GMV, is mainly descriptive and needs further investigation, although it has been reported that amyloid is the preliminary pathogenesis. Thirdly, this is a cross-sectional study. Although we try to use the subjects with different disease stages to depict the AD continuum, a further longitudinal study should be done. Moreover, due to the small overlap between the available Tau PET and PHS data, we cannot include the Tau PET in the current study. This may partially limit our understanding of the genetic effect on AD pathology and should be further explored in future studies. Finally, the current study only focused on PHS, and further study should consider other polygenic risk scores (PRS), like wider AD-PRSs (including genetic variants at *P*-value threshold < 0.5) (Escott-Price et al., 2015) or derive a PRS by ourselves according to the scientific design.

## Conclusion

Based on a data-driven, supervised-learning fusion method, our study revealed that subject-specific PHS was linked with multi-facets of the brain, including function, pathological deposition, and neurodegeneration. The multimodal brain abnormalities were further correlated with cognition. The identified atypical brain regions spatially involved DMN, ECN, as well as visuospatial network and showed progressive changes with the development of AD. This work expands our understanding of how genetic risk factors in AD contribute to brain impairments and provides insight into how plausible genetic risk factors may influence the pathophysiology of AD. Moreover, different disease stages show different sensitivity to different imaging parameters, suggesting that specific neuroimaging methods should be selected according to the disease stages during the clinical diagnosis and treatment.

## Data Availability Statement

Publicly available datasets were analyzed in this study. This data can be found here: The datasets generated and analyzed during the current study are available in the ADNI study. More details in www.adni-info.org.

## Ethics Statement

All procedures performed in studies involving human participants were in accordance with the ethical standards of the institutional and national research committee and with the 1964 Helsinki declaration and its later amendments or comparable ethical standards. Written informed consent was obtained from all participants and authorized representatives, and the study partners before any protocol-specific procedures were carried out in the ADNI study. More details in http://www.adni-info.org. The patients/participants provided their written informed consent to participate in this study.

## Author Contributions

KL designed the study and wrote the first draft of the manuscript. ZF and SQ analyzed the MRI data and wrote the protocol. XL, PH, and VC assisted with study design and interpretation of findings. QZ collected clinical and MRI data. XX modified the expression and grammar thoroughly. All authors have contributed to and approved the final manuscript.

## Conflict of Interest

The authors declare that the research was conducted in the absence of any commercial or financial relationships that could be construed as a potential conflict of interest.

## Publisher’s Note

All claims expressed in this article are solely those of the authors and do not necessarily represent those of their affiliated organizations, or those of the publisher, the editors and the reviewers. Any product that may be evaluated in this article, or claim that may be made by its manufacturer, is not guaranteed or endorsed by the publisher.

## Footnotes

1
http://adni.loni.usc.edu/

2
https://ida.loni.usc.edu

3
www.rfmri.org/dpasfa

4
www.fil.ion.ucl.ac.uk/spm

5
https://trendscenter.org/software/fit/
