# Supplementary material for: Polygenic Hazard Score Associated Multimodal Brain Networks Along the Alzheimer’s Disease Continuum
Source: Front Aging Neurosci. 2021 Sep 3;13:725246. doi: 10.3389/fnagi.2021.725246 (PMC8446666; doi:10.3389/fnagi.2021.725246)
Supplement: Supplementary file 1 [file Data_Sheet_1.docx]

# Polygenic hazard score associated multimodal brain networks along the Alzheimer's disease continuum

# Kaicheng Li ^1, 2^†, Zening Fu^1^†, Shile Qi^3^†, Xiao Luo^2^, Qingze Zeng^2^, Xiaopei Xu^2^, Peiyu Huang^2^, Minming Zhang*^2^, Vince D. Calhoun^1, 4, 5^, and the Alzheimer's Disease Neuroimaging Initiative^6^

^1^Tri-institutional Center for Translational Research in Neuroimaging and Data Science (TReNDS): Georgia State University, Georgia Institute of Technology, Emory University, Atlanta, Georgia, USA

^2^Department of Radiology, 2nd Affiliated Hospital of Zhejiang University School of Medicine, China

^3^Department of Computer Science and Engineering, Nanjing University of Aeronautics and Astronautics, Nanjing 211106, China

^4^Department of Psychology, Computer Science, Neuroscience Institute, and Physics, Georgia State University, Atlanta, Georgia.

^5^Department of Electrical and Computer Engineering, Georgia Institute of Technology, Atlanta, Georgia

^6^Data used in the preparation of this article were obtained from the Alzheimer's disease Neuroimaging Initiative (ADNI) database (http://www.adni.loni.usc.edu). As such, the investigators within the ADNI contributed to the design and implementation of ADNI and provided data but did not participate in analysis or writing of this report. A complete listing of ADNI investigators can be found at <http://adni.loni.usc.edu/wp-content/up> loads/how to apply/ADNI Acknowledgement List.pdf.

**Corresponding to:**

*Prof. Minming Zhang, MD, PhD

Department of Radiology, The 2nd Affiliated Hospital of Zhejiang University, School of Medicine, No.88 Jie-fang Road, Shang-cheng District, Hangzhou, China, 310009; Phone: 86-0571-87315255; Fax: 86-0571-87315255; Email address: zhangminming@zju.edu.cn

**Supplementary Material 1.** **Alzheimer's disease neuroimaging and initiative**

The Alzheimer's disease neuroimaging initiative (ADNI) database was initially launched in 2003 (ADNI-1) by the National Institute on Aging (NIA), the Food and Drug Administration (FDA), the National Institute of Biomedical Imaging and Bioengineering (NIBIB), and additional recruitment was made through ADNI-GO in 2009, ADNI-2 in 2010 and ADNI-3 in 2016. The primary goal of ADNI has been to identify serial magnetic resonance imaging (MRI), positron emission tomography (PET), biomarkers, and genetic characteristics that would support the early detection and tracking of AD, and improved clinical trial design. For up-to-date information, see <http://www.adni-info.org>.

**Supplementary Material 2. MRI and PET acquisition**

The structural images were obtained based on 3D Magnetization Prepared Rapid Acquisition Gradient Echo (MPRAGE) T1 weighted sequence, with the following parameters: voxel size=1.1 × 1.1 × 1.2 mm^3^; echo time (TE)=2.98 ms; inversion time (TI)=900 ms; repetition time (TR)=2300 ms; 170 sagittal slices; within plane FOV=256 × 240 mm^2^.

The resting-state functional MRI (rsfMRI) images were obtained using an echo-planar imaging sequence, with the following parameters: TE=30 ms; TR=3000 ms; the number of slices=48; slice thickness=3.3 mm; spatial resolution=3.31×3.31×3.31 mm^3^. According to the ADNI scanning protocol, all subjects were instructed to open their eyes, focusing on a cross, and keep at rest calmly during the scan.

The ^18^F-florbetapir positron emission tomography ([^18^F]-AV45 PET) in ADNI were acquired according to a standard dynamic 50–70 min protocol after the intravenous bolus injection of 370 ± 37 MBq of [^18^F]-AV45 (More detailed information can be found on the ADNI website: <http://adni.loni.usc.edu/methods/>). Then, the acquired [^18^F]-AV45 PET image in ADNI undergoes standardized preprocessing correction steps to increase data uniformity: 1) motion correction by co-registration of single five-minute frames; 2) time frame averaging (50–70 min p.i.); 3) co-registration of longitudinal data to the baseline scan and reorientation in a standardized 160 × 160 × 96 matrices with 1.5 mm cubic voxels; 4) smoothing with a scanner-specific filter function to an isotropic resolution of 8 mm. All [^18^F]-AV45 PET images in the current study were downloaded from the ADNI database in the most fully preprocessed format ("AV45 Coreg, Avg, Std Img and Vox Siz, Uniform Resolution" for PET data).

**Supplementary Material 3. Flowchart of subjects inclusion**

We identified 248 subjects who had the T1-weighted structural scan, [^18^F]-AV45 PET, rsfMRI, PHS, and comprehensive neuropsychological assessments from ADNI GO, ADNI 2, and ADNI 3 database before 2020 May. All the cognitive status and multimodal imaging data were obtained from the same visit. Notably, according to the ADNI protocol, once the visit begins, all imaging and clinical/cognitive assessments must take place within the next 2 weeks. Detailed inclusions were: firstly, we included the subjects with PHS, T1-weighted structural scan, and rsfMRI. Here, imaging data are required to be obtained at the same timepoint. Secondly, we further select time-matched PET data. Notably, if some subjects did not have time-matched PET at baseline, then the longitudinal multimodal data will be chosen. Accordingly, we got 248 subjects with PHS and time-matched multimodal imaging (T1-weighted structural scan, [18F]-AV45 PET, and rsfMRI). After careful screening, 26 subjects were excluded due to different rsfMRI scan parameters or bad image quality; 14 subjects were excluded due to abnormal cognitive performance; 9 subjects were excluded due to large headmotion (subjects with more than 3 mm maximum displacement in any of the x, y, or z directions or 3° of any angular motion were discarded); 12 subjects were excluded due to rsfMRI/ [^18^F]-AV45 PET image analysis failure. Finally, we included 187 subjects, including 88 healthy control (HC), 77 subjects with mild cognitive impairment (MCI), and 22 Alzheimer's disease (AD) patients (Figure s1). Notably, the current classification of phenotypes was made based on the ADNI diagnosis at the specific visit when the MRI was retrieved. Detailed diagnosis information can be downloaded from the ADNI database, “study data>diagnosis> Diagnostic Summary [ADNI1,GO,2,3]” sheet name: DXSUM_PDXCONV_ADNIALL．

The HCs were defined as subjects who had a Clinical Dementia Rating scale (CDR) score of 0, a Mini-Mental State Examination (MMSE) between 24 and 30 (inclusive), Wechsler Memory Scale Logical Memory, WMS-LM, delay recall performance ≥ 9 for subjects with 16 or more years of education; ≥ 5 for subjects with 8 – 15 years of education; and ≥ 3 for 0 – 7 years of education; non clinical depression (Geriatric Depression Scale-15, GDS-15 score < 6) and absence of dementia [1]. MCI patients were defined as subjects who had preserved activities of daily living, non-dementia, and objective cognitive impairments, as shown on the delayed recall test of the WMS-LM as well as a CDR score of 0.5 [2]. AD patients were defined as subjects who had MMSE of ≤ 26, CDR of ≥ 0.5, as well as met the NINCDS/ADRDA criteria for probable AD [3]. Exclusion criteria are listed below: 1) significant medical, neurological, and psychiatric illness; 2) head trauma history; 3) use of non-AD-related medication known to influence cerebral function; 4) clinical depression; 5) alcohol or drug abuse.

Figure s1. Flowchart of subjects inclusion


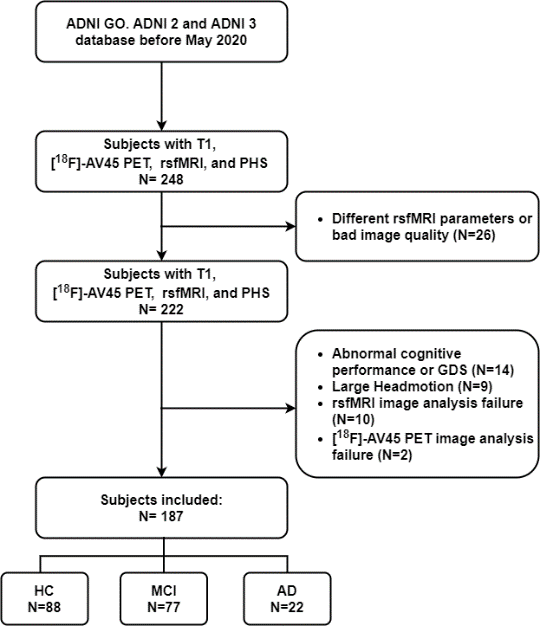


**Abbreviation:** ADNI: the Alzheimer's disease neuroimaging initiative (ADNI); [^18^F]-AV45 PET：^18^F-florbetapir positron emission tomography; rsfMRI: rest-state functional magnetic resonance imaging; PHS: polygenic hazard score; GDS: geriatric depression scale; HC: healthy control; MCI: mild cognitive impairment; AD: Alzheimer's disease

**Supplementary Material 4. Detailed information about the multiple composite cognitive scores**

In the current study, we included comprehensive cognitive scores as the observation indexes, including the assessment of memory (ADNI memory composite score (ADNI-MEM)) [4], executive function (ADNI executive function composite score (ADNI-EF)) [5], language function (ADNI language function composite score (ADNI-LAN)), and visuospatial function (ADNI visuospatial function composite score (ADNI-VS)). All these four indexes are derived from data from the ADNI neuropsychological battery and are useful in reflecting cognition.

Briefly, ADNI-MEM is a validated composite memory score derived from the longitudinal Rey Auditory Verbal Learning Test (AVLT), AD Assessment Scale (ADAS)–cognition, Mini-Mental State Examination (MMSE), and Wechsler Memory Scale Logical Memory (WMS-LM). Here, modern psychometric approaches were applied. ADNI-EF is a validated composite executive function score derived from WAIS-R Digit Symbol Substitution, Digit Span Backwards, Trail-Making Test part A (TMT-A), and TMT B, Category Fluency, and Clock-Drawing Test (CDT). To be specific, item response theory methods were applied. The criteria for model fit were the confirmatory fit index (CFI), the Tucker Lewis Index (TLI), and the root mean squared error of approximation (RMSEA), where criteria for excellent fit include CFI>0.95, TLI >0.95, and RMSEA<0.05 [6]. Similarly, ADNI-LAN is derived from several language-related tests from ADNI neuropsychological battery, MMSE, ADAS-cognitive behavior, and Montreal Cognitive Assessment (MoCA). ADNI-VS is derived from clock copy, the constructional praxis from ADAS-cognitive behavior, and copy design from MMSE. Here, item response theory was used to derive composite measures (ADNI-LAN, and ADNI-VS). More detailed information can be found in <http://www.adni-info.org>.

**Supplementary Material 5.** **Anatomical information of the identified regions in the joint component**

As for fALFF, higher PHS is linked with decreased values in fALFF in the precuneus, inferior parietal lobule (IPL), and middle temporal gyrus (MTG). Moreover, higher PHS is associated with increased values in fALFF in the hippocampus, parahippocampal gyrus, and frontal regions (Table s1).

As for gray matter volume (GMV), higher PHS is linked with decreased values in GMV in the precuneus, IPL, temporal gyrus (including parahippocampal gyrus and hippocampus), and basal ganglia nucleus. Moreover, higher PHS is linked with increased GMV in the frontal and occipital regions (Table s2).

As for amyloid SUVR, higher PHS is linked with widespread increased values in amyloid SUVR, especially in the precuneus, IPL, posterior cingulate cortex (PCC), as well as temporal and frontal regions (Table s3).

All three features commonly involved the precuneus, IPL, PCC, and temporal regions, which consist of essential components of the default mode network (DMN). Moreover, the involved frontal and occipital regions indicate the impairments of the executive control network and visuospatial network, respectively.

Table s1. Anatomical information of the identified joint component of fALFF

| **Area** | **Brodmann Area** | **Volume (cc)** | **Random effects: Max Value (x, y, z)** |
| --- | --- | --- | --- |
| **Negative** | | | |
| Precuneus | 7, 19 | 4.4/1.0 | 3.2 (-24, -56, 47)/3.0 (12, -58, 58) |
| Cuneus | 18, 19 | 0.2/0.0 | 2.2 (-15, -77, 26)/-999.0 (0, 0, 0) |
| Inferior Parietal Lobule | 40 | 4.3/0.3 | 3.2 (-56, -36, 35)/2.2 (50, -59, 44) |
| Superior Parietal Lobule | 7 | 2.4/0.7 | 3.1 (-27, -62, 47)/2.7 (15, -61, 56) |
| Supramarginal Gyrus | / | 0.2/0.2 | 2.1 (-53, -42, 35)/2.3 (48, -45, 35) |
| Middle Temporal Gyrus | / | 0.1/0.0 | 2.0 (-33, -66, 28)/-999.0 (0, 0, 0) |
| Insula | 13 | 0.1/0.8 | 2.0 (-39, -20, 9)/2.8 (42, -2, 11) |
| **Positive** | | | |
| Parahippocampal Gyrus | 27, 28, 30, 34, 35 | 4.2/1.9 | 3.9 (-27, -24, -9)/3.5 (27, -21, -9) |
| Uncus | 28, 34 | 0.7/0.3 | 2.6 (-15, -7, -20)/2.4 (18, -1, -20) |
| Superior Temporal Gyrus | 22, 38, 41, 42 | 2.7/1.0 | 2.8 (-36, 7, -26)/2.5 (24, 13, -26) |
| Inferior Frontal Gyrus | 45, 47 | 1.4/0.4 | 2.9 (-18, 8, -18)/2.6 (27, 16, -21) |
| Middle Frontal Gyrus | 6, 11 | 0.9/0.1 | 2.8 (-42, 0, 53)/2.1 (42, 11, 55) |
| Superior Frontal Gyrus | 6, 8, 9 | 2.0/0.0 | 2.6 (-9, 46, 45)/-999.0 (0, 0, 0) |
| Medial Frontal Gyrus | 6, 25 | 0.6/0.4 | 2.5 (-15, 11, -18)/2.6 (3, -6, 50) |
| Lingual Gyrus | 17, 18, 19 | 0.1/0.6 | 2.2 (-15, -47, 2)/2.8 (6, -88, -13) |
| Subcallosal Gyrus | 13, 47 | 0.4/0.1 | 2.8 (-15, 11, -13)/2.3 (15, 11, -13) |
| Cingulate Gyrus | 24, 32 | 0.6/1.0 | 2.7 (-3, -7, 28)/2.5 (6, 13, 35) |

Abbreviation: fALFF: fractional amplitude of low frequency fluctuations

Table s2. Anatomical information of the identified joint component of GMV

| Area | Brodmann Area | volume (cc) | random effects: Max Value (x, y, z) |
| --- | --- | --- | --- |
| **Negative** | | | |
| Parahippocampal Gyrus | 19, 28, 34, 35, 36, 37 | 11.5/11.2 | 6.7 (-24, -7, -17)/6.9 (24, -7, -17) |
| Uncus | 20, 28, 34, 36, 38 | 5.1/4.7 | 6.3 (-24, -4, -20)/6.5 (24, -4, -20) |
| Inferior Temporal Gyrus | 19, 20, 21, 37 | 8.5/8.7 | 6.2 (-59, -21, -19)/6.9 (59, -27, -19) |
| Middle Temporal Gyrus | 20, 21, 22, 37, 38, 39 | 17.0/14.7 | 6.2 (-65, -21, -12)/5.9 (62, -24, -14) |
| Superior Temporal Gyrus | 13, 21, 22, 38, 39, 42 | 11.4/15.2 | 4.1 (-27, 7, -31)/5.2 (65, -34, 16) |
| Fusiform Gyrus | 20, 36, 37 | 4.4/6.7 | 5.2 (-56, -19, -24)/5.9 (59, -19, -24) |
| Inferior Parietal Lobule | 7, 39, 40 | 6.3/8.4 | 4.6 (-59, -39, 32)/5.1 (56, -36, 32) |
| Precuneus | 7, 19 | 2.4/1.9 | 3.4 (-33, -74, 37)/4.2 (6, -68, 42) |
| Supramarginal Gyrus | 40 | 3.0/3.0 | 4.3 (-59, -42, 30)/4.7 (53, -36, 35) |
| Superior Parietal Lobule | 7 | 1.2/0.6 | 4.5 (-27, -65, 50)/2.7 (36, -68, 45) |
| Angular Gyrus | 39 | 0.4/0.1 | 2.6 (-33, -74, 31)/2.4 (36, -71, 31) |
| Postcentral Gyrus | 1, 2, 3, 40, 43 | 2.1/4.1 | 3.5 (-59, -22, 20)/4.8 (62, -28, 18) |
| Precentral Gyrus | 4, 6 | 4.2/3.6 | 4.1 (-39, -6, 56)/3.4 (33, -9, 61) |
| Thalamus | / | 4.9/6.0 | 6.9 (-9, -17, 9)/6.1 (12, -17, 9) |
| Caudate | / | 0.1/0.1 | 2.3 (-12, 17, -6)/2.5 (36, -27, -6) |
| Insula | 13 | 2.2/2.8 | 5.6 (-36, -6, -2)/5.4 (36, 0, 0) |
| Lentiform Nucleus | / | 2.1/1.6 | 4.6 (-27, 3, 0)/4.5 (27, 6, 0) |
| **Positive** | | | |
| Middle Frontal Gyrus/Superior Frontal Gyrus/Medial Frontal Gyrus | 6, 8, 9, 10, 11, 46 | 5.1/6.5 | 4.1 (-36, 8, 41)/3.7 (30, 40, 34) |
| Inferior Frontal Gyrus | 6, 45 | 1.2/0.4 | 3.7 (-36, 7, 33)/2.6 (39, 5, 33) |
| Middle Occipital Gyrus | 18, 19, 37 | 1.3/0.7 | 3.9 (-39, -64, 3)/2.8 (39, -67, 9) |
| Calcarine gyrus | 7, 17, 18, 19, 23, 30 | 8.3/12.3 | 3.9 (-15, -69, 15)/5.2 (12, -72, 15) |
| Lingual Gyrus | 17, 18, 19 | 4.2/7.1 | 3.7 (-15, -52, 5)/4.3 (9, -87, 4) |

Abbreviation: GMV: gray matter volume

Table s3. Anatomical information of the identified joint component of amyloid SUVR

| **Area** | **Brodmann Area** | **volume (cc)** | **random effects: Max Value (x, y, z)** |
| --- | --- | --- | --- |
| **Positive** | | | |
| Precuneus | 7, 31, 39 | 2.3/2.0 | 2.5 (-6, -48, 33)/2.5 (6, -48, 30) |
| Cingulate Gyrus/Posterior Cingulate | 23, 31 | 1.7/1.8 | 2.5 (-6, -45, 35)/2.6 (9, -45, 35) |
| Supramarginal Gyrus/Inferior Parietal Lobule | 40 | 0.7/1.5 | 2.2 (-53, -54, 30)/2.5 (56, -48, 25) |
| Angular Gyrus | 39 | 0.4/0.8 | 2.2 (-48, -65, 31)/2.4 (48, -63, 31) |
| Superior Temporal Gyrus | 13, 22, 39 | 1.5/3.1 | 2.2 (-50, -49, 11)/2.4 (50, -38, 7) |
| Inferior Temporal Gyrus/Fusiform Gyrus | 19, 20, 21, 36, 37 | 3.0/4.0 | 2.7 (-53, -13, -27)/2.9 (59, -19, -22) |
| Middle Temporal Gyrus | 19, 20, 21, 22, 37, 39 | 6.0/6.8 | 2.5 (-56, -53, -10)/2.6 (59, -41, -13) |
| Inferior Frontal Gyrus | 10, 46, 47 | 0.5/0.3 | 2.2 (-42, 38, 6)/2.2 (48, 37, -12) |
| Middle Frontal Gyrus/Superior Frontal Gyrus/Medial Frontal Gyrus/Rectal Gyrus | 9, 10, 11 | 3.4/2.4 | 2.4 (-24, 55, -10)/2.5 (30, 55, -10) |
| Anterior Cingulate | / | 0.1/0.0 | 2.0 (-6, 44, -2)/-999.0 (0, 0, 0) |
| Middle Occipital Gyrus | 19, 37 | 1.3/0.4 | 2.3 (-48, -67, 3)/2.4 (53, -59, -5) |

Abbreviation: SUVR: standard uptake value ratios

**Supplementary Material 6. Correlation between features and cognitive scores**

Table s4 showed the Pearson correlation between the identified components and cognitive scores across subjects.

To show the distinct association with cognition, we further performed the correlation analysis within every group (HC, MCI, and AD). Results showed the most significant association between memory and GMV, especially in subjects in MCI/AD. Detailed information was listed in Table s5.

To remove the possible effect of covariates (age, gender, and education level), we further performed the partial correlation on the loadings of the identified joint component and cognitive function. As shown in Table s6, results are largely consistent with the Pearson correlation results. Such significant associations between the multimodal features and cognition in AD-continuum subjects indicate the robustness of the identified joint component in indexing the cognitive declines.

Table s4. Correlation between loadings of the identified components and cognitive scores across subjects.

| **Cognitive scores** | **fALFF** | | **GMV** | | **SUVR** | |
| --- | --- | --- | --- | --- | --- | --- |
|  | *r* | *p* | *r* | *p* | *r* | *p* |
| **Memory** | -0.20^*^ | 0.007 | -0.40^*^ | <0.001 | -0.29^*^ | <0.001 |
| **Executive function** | -0.22^*^ | 0.003 | -0.23^*^ | 0.002 | -0.27^*^ | <0.001 |
| **Language** | -0.20^*^ | 0.005 | -0.27^*^ | <0.001 | -0.22^*^ | 0.002 |
| **Visuospatial function** | -0.17^*^ | 0.017 | -0.13 | 0.071 | -0.22^*^ | 0.002 |

* P<0.05, FDR corrected.

Abbreviation: fALFF: fractional amplitude of low frequency fluctuations, GMV: gray matter volume; SUVR: standard uptake value ratios

Table s5. Correlation between loadings of the identified components and cognitive scores within every group (HC, MCI, and AD)

| Control Variables | Cognitive scores | fALFF | | GMV | | Amyloid SUVR | |
| --- | --- | --- | --- | --- | --- | --- | --- |
|  |  | *r* | *p* | *r* | *p* | *r* | *p* |
| HC | **Memory** | 0.02 | 0.875 | -0.10 | 0.351 | -0.02 | 0.831 |
|  | **Executive function** | -0.19 | 0.079 | 0.04 | 0.700 | -0.14 | 0.205 |
|  | **Language** | -0.01 | 0.900 | 0.02 | 0.822 | 0.02 | 0.853 |
|  | **Visuospatial function** | 0.05 | 0.622 | -0.06 | 0.599 | **-0.21** | **0.045** |
| MCI | **Memory** | -0.18 | 0.112 | **-0.28** | **0.013** | -0.16 | 0.171 |
|  | **Executive function** | -0.19 | 0.109 | -0.14 | 0.220 | -0.16 | 0.163 |
|  | **Language** | -0.14 | 0.216 | -0.15 | 0.191 | -0.13 | 0.246 |
|  | **Visuospatial function** | -0.14 | 0.228 | 0.04 | 0.702 | -0.14 | 0.238 |
| AD | **Memory** | -0.08 | 0.725 | **-0.62** | **0.002** | -0.31 | 0.167 |
|  | **Executive function** | 0.06 | 0.798 | -0.02 | 0.932 | -0.01 | 0.977 |
|  | **Language** | -0.25 | 0.259 | -0.19 | 0.397 | -0.05 | 0.816 |
|  | **Visuospatial function** | **-0.45** | **0.037** | -0.01 | 0.983 | 0.09 | 0.702 |
| MCI+AD | **Memory** | -0.19 | 0.057 | **-0.48** | **0.000** | **-0.29** | **0.003** |
|  | **Executive function** | -0.15 | 0.145 | **-0.26** | **0.010** | **-0.22** | **0.029** |
|  | **Language** | **-0.20** | **0.048** | **-0.30** | **0.003** | **-0.21** | **0.036** |
|  | **Visuospatial function** | **-0.24** | **0.019** | -0.10 | 0.333 | -0.16 | 0.117 |

Abbreviation: HC: healthy control; MCI: mild cognitive control; AD: Alzheimer's disease; fALFF: fractional amplitude of low frequency fluctuations, GMV: gray matter volume; SUVR: standard uptake value ratios

Table s6. Correlation between multimodal features and cognitive scores by regressing out variables

| Control Variables | Cognitive scores | fALFF | | GMV | | Amyloid SUVR | |
| --- | --- | --- | --- | --- | --- | --- | --- |
|  |  | *r* | *p* | *r* | *p* | *r* | *p* |
| Age | **Memory** | -0.16* | 0.032 | -0.41* | <0.001 | -0.26* | 0.001 |
|  | **Executive function** | -0.20* | 0.006 | -0.26* | <0.001 | -0.23* | 0.002 |
|  | **Language** | -0.15 | 0.045 | -0.27* | <0.001 | -0.16* | 0.033 |
|  | **Visuospatial function** | -0.12 | 0.122 | -0.13 | 0.093 | -0.19* | 0.009 |
| Age+Gender | **Memory** | -0.15 | 0.042 | -0.42* | <0.001 | -0.28* | <0.001 |
|  | **Executive function** | -0.20* | 0.007 | -0.26* | <0.001 | -0.24* | 0.001 |
|  | **Language** | -0.15 | 0.047 | -0.27* | <0.001 | -0.16* | 0.029 |
|  | **Visuospatial function** | -0.12 | 0.112 | -0.13 | 0.091 | -0.19* | 0.011 |
| Age+Gender+Education | **Memory** | -0.10 | 0.166 | 0.44* | <0.001 | -0.29* | <0.001 |
|  | **Executive function** | -0.13 | 0.076 | 0.29* | <0.001 | -0.25* | 0.001 |
|  | **Language** | -0.09 | 0.227 | 0.30* | <0.001 | -0.17* | 0.026 |
|  | **Visuospatial function** | -0.08 | 0.310 | 0.14 | 0.061 | -0.19* | 0.011 |

* P<0.05, FDR corrected.

Abbreviation: fALFF: fractional amplitude of low frequency fluctuations, GMV: gray matter volume; SUVR: standard uptake value ratios

**Supplementary Material 7.**

Figure s2. Group differences of the loading parameters of the identified joint component by including the age, gender, and education as covariables.


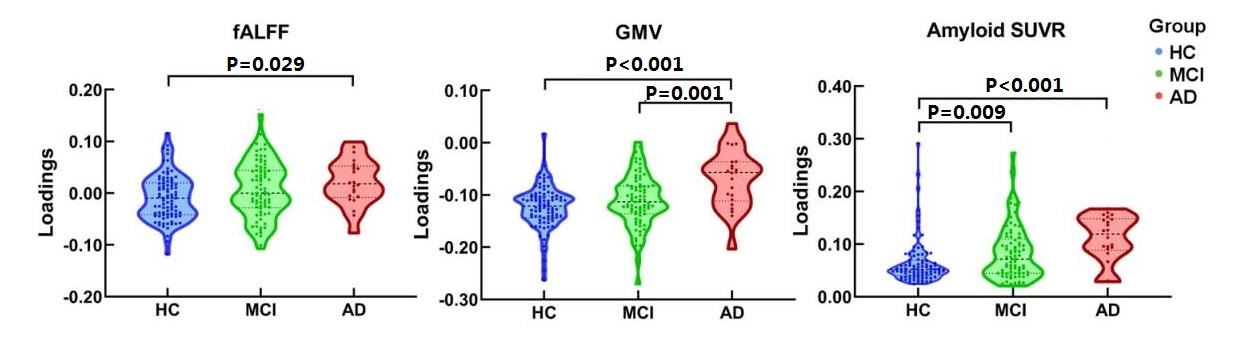


Abbreviation: fALFF: fractional amplitude of low frequency fluctuations; GMV: gray matter volume; SUVR: standard uptake value ratios; HC: healthy control; MCI: mild cognitive decline; AD: Alzheimer's disease

**Supplementary Material 8.**

APOE ε4 allele is the primary susceptibility gene for LOAD [7]. Thus, we tried to supplementarily identify the role of APOE alone and the other SNPs on the brain changes from the following three aspects: 1) we reduced the effect of APOE by including APOE status as a covariate during the group comparisons and correlation analysis; 2) similar to another study focusing on the PHS [8], we performed totally the same fusion analysis in APOE ε3 homozygous individuals, whose AD risk was resulted by the SNPs beyond APOE; 3) we explored the effect of APOE ε4 and ε2 on three neuroimaging features and qualitatively compared with that of PHS respectively to show the possible effect difference of two genetic indexes. Overall analysis showed that the effect of genetic risk scores with and without APOE showed both similarities and differences, suggesting that both APOE and other SNPs play an important role in the occurrence and development of AD.

Firstly, similar to previous PHS-related studies [9], we reduced the possible effect of APOE by including APOE status as a covariate. To be specific, we examined the group differences of loadings using the GLM: loadings of three neuroimaging features (fALFF, GMV, and [^18^F]-AV45 PET SUVR) as the dependent variable; group as a categorical predictor, APOE status (binarized as having at least one copy of the 4 alleles versus none) as covariable to control the effect of APOE. Results keep largely the same as the original results (Figure s3).

Figure s3. Group differences of the loading parameters of the identified joint component by including the APOE status as covariable.


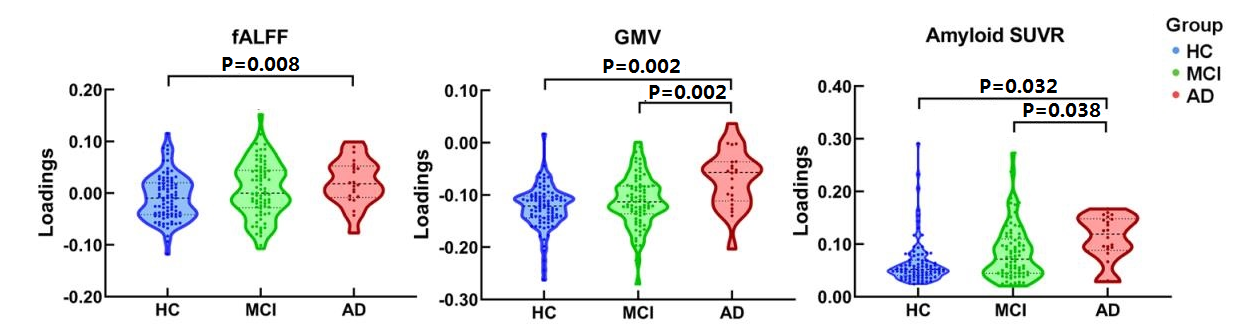


Abbreviation: fALFF: fractional amplitude of low frequency fluctuations; GMV: gray matter volume; SUVR: standard uptake value ratios; HC: healthy control; MCI: mild cognitive decline; AD: Alzheimer's disease

Secondly, we performed totally the same fusion analysis in APOE ε3 homozygous individuals (N=122, HC=74, MCI=38, AD=10) (Table s7). The AD risk of APOE ε3 homozygous individuals results from the SNPs beyond APOE and thus work as the ideal subjects to explore the effect of PHS without APOE. This method is similar to another study focusing on the PHS [8] and successfully proves the effect of effects of AD genetic risk beyond APOE.

To be specific, MCCAR+ jICA was used to perform the 3-way fusion analysis. One joint component was identified that was correlated with PHS in the APOE ε3 homozygous individuals. The resulting spatial maps were Z-transformed and visualized at |Z|>2 in Figure s4. Along AD-continuum, PHS was correlated with 1) decreased fALFF in the inferior parietal lobule (IPL), and middle frontal gyrus, while increased fALFF in the temporal region and supplementary motor area (SM); 2) decreased GMV in the subcortical regions, occipital regions, and temporal region, while increased GMV in the precuneus, IPL, and frontal region; 3) increased amyloid SUVR in the IPL, and PCC, while decreased amyloid SUVR in the subcortical regions. Furthermore, the loadings of ICs were positively correlated with PHS (r = 0.26, p = 0.004 for fALFF; r = 0.22, p = 0.01 for GMV; r = 0.21, p = 0.02 for amyloid SUVR). Significant differences in loadings of amyloid SUVR among groups were also observed. To be specific, both MCI and AD showed higher loadings of amyloid SUVR when compared to HC; notably, AD showed higher loading of amyloid SUVR than MCI. The overall trend keeps the same with original results, although the effect is relatively weaker. The possible reasons may be the relatively small sample size and the relatively weak effect of the other SNPs.

Conclusively, the identified brain regions show both similarity and differences with our results using PHS as the reference, proving that PHS has some effect beyond APOE.

Table s7. Demographic information

| **Demographic characteristics** | **HC** | **MCI** | **AD** | **F-value/X^2^** | **Sig** |
| --- | --- | --- | --- | --- | --- |
|  | **N=74** | **N=38** | **N=10** |  |  |
| Age | 77.49±6.06 | 79.46±7.48 | 83.85±8.21 | 4.39 | 0.015b |
| Gender (F/M) | 33/41 | 16/22 | 5/5 | 0.21 | 0.90 |
| Education | 16.61±2.71 | 16.29±2.68 | 15.50±3.34 | 0.77 | 0.46 |
| GDS | 0.80±1.07 | 1.42±1.22 | 2.20±1.69 | 8.23 | <0.001ab |
| PHS | -0.25±0.22 | -0.26±0.26 | -0.06±0.17 | 3.32 | 0.04bc |
| **Cognitive Scores** | | | | | |
| MMSE | 29.01±1.14 | 28.47±1.48 | 19.10±5.30 | 121.47 | <0.001bc |
| CDR global | 0.00±0.00 | 0.50±0.00 | 1.00±0.41 | 500.06 | <0.001abc |
| CDR sum | 0.03±0.13 | 1.36±0.99 | 5.55±2.97 | 142.39 | <0.001abc |
| ADNI_MEM | 1.10±0.64 | 0.35±0.52 | -0.76±0.61 | 51.83 | <0.001abc |
| ADNI_EF | 1.10±0.81 | 0.59±0.84 | -0.95±1.03 | 25.67 | <0.001abc |
| ADNI_LAN | 0.98±0.67 | 0.50±0.82 | -1.15±1.25 | 34.09 | <0.001abc |
| ADNI_VS | 0.16±0.63 | 0.09±0.74 | -1.07±1.13 | 13.15 | <0.001bc |

Data are presented as means ± standard deviations.

Abbreviation: HC: healthy control; MCI: mild cognitive impairment; AD: Alzheimer's disease; APOE: apolipoprotein; GDS: geriatric depression scale; PHS: polygenic hazard score; MMSE, Mini-Mental State Examination; CDR: clinical dementia rating; ADNI-MEM: the composite scores for memory in ADNI; ADNI-EF: the composite scores for executive function in ADNI; ADNI-LAN: the composite scores for language in ADNI; ADNI-VS: the composite scores for visuospatial function in ADNI

a-c: post-hoc analysis further revealed the source of ANOVA difference (a: HC vs. MCI; b: HC vs. AD; c: MCI vs. AD) (p<0.05, significant difference between groups).

Figure s4. The identified joint component in APOE ε3 homozygous individuals.


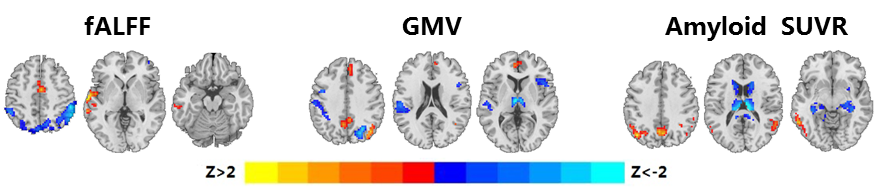


The spatial maps are visualized at |Z|>2, where the positive Z-values (red regions) mean higher fALFF, GMV, and more amyloid deposition, and negative Z-values (blue regions) indicate decreased fALFF, GMV, and less amyloid deposition.

Abbreviation: fALFF: fractional amplitude of low frequency fluctuations; GM: gray matter; SUVR: standard uptake value ratios

Thirdly, APOE related multimodal changes is very important. However, algorithmically, the "MCCAR+jICA" model used Pearson correlation to maximize the PHS-modality correlation, while "APOE" is a categorical variable (Pearson correlation is limited to continue variables). So, it is not allowed to repeat the data fusion analysis using the APOE status as the reference due to the limitation of "MCCAR+jICA" model.

To further address the reviewers' concern, we explored the effect of APOE ε4 and ε2 on three neuroimaging features and qualitatively compared with that of PHS respectively to show the possible effect difference of two genetic indexes. To be specific, we performed two-sample T-test between APOE ε4, ε2, and ε3 (APOE ε4 VS. APOE ε3, and APOE ε2 VS. APOE ε3) to explore the effect of APOE ε4 and ε2 on three neuroimaging features (fALFF, GMV, and voxel-wise amyloid SUVR). We set the threshold at p < 0.05 at the voxel level, p < 0.05 at the cluster level, with Gaussian random field correction (GRF) corrected. Moreover, we performed the voxel-wise correlation analysis between the PHS and three neuroimaging features in every subgroup (APOE ε2, ε3, ε4). Combining the above analysis, we can qualitatively explore the possible different effects of APOE and PHS.

As for voxel-wise amyloid SUVR, the two-sample t-test showed that APOE ε4 carriers featured significantly increased amyloid deposition involving the whole cortex. The correlation analysis in APOE ε4 carriers showed a significant association between PHS and increased amyloid deposition in the middle frontal gyrus (Figure s5). Although no significant difference in the APOE ε2 analysis was found, they did show a trend of decreased amyloid deposition, indicating its protective effect from AD pathology. Our results showed that APOE and PHS have similar effect targets but different strengths on the amyloid.

Figure s5. The effect of APOE ε4 on amyloid SUVR


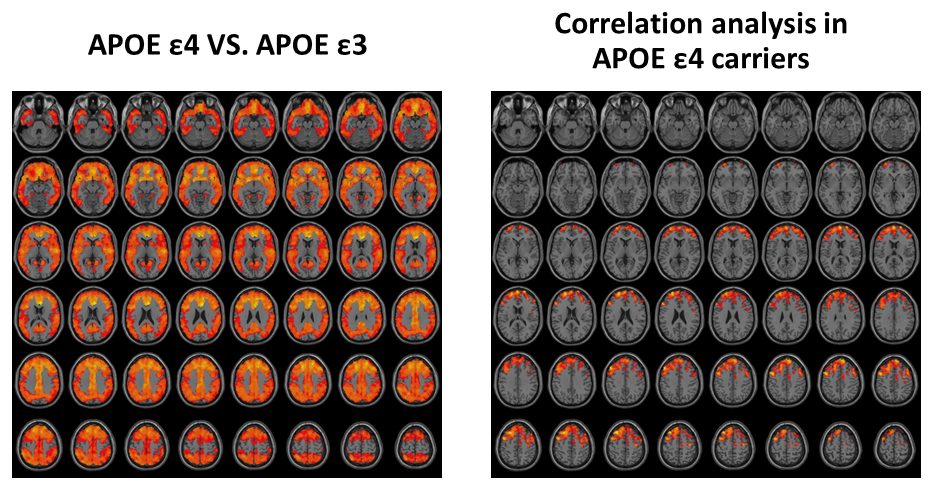


Abbreviation: APOE: apolipoprotein E; SUVR: standard uptake value ratios

As for fALFF analysis, the two-sample t-test showed that APOE ε4 carriers featured significantly decreased fALFF in the precuneus and increased fALFF in the middle cingulate gyrus; the correlation analysis in APOE ε4 carriers showed a significant increase in the middle frontal gyrus (Figure s6). The two-sample t-test on the APOE ε2 carriers found the increased fALFF in superior temporal and postcentral regions when compared to APOE ε3. The correlation analysis in APOE ε2 carriers showed that PHS correlates with decreased fALFF in the precuneus (Figure s7). Conclusively, qualitative analysis shows that APOE and PHS have relatively different effects on brain function.

Figure s6. The effect of APOE ε4 on fALFF


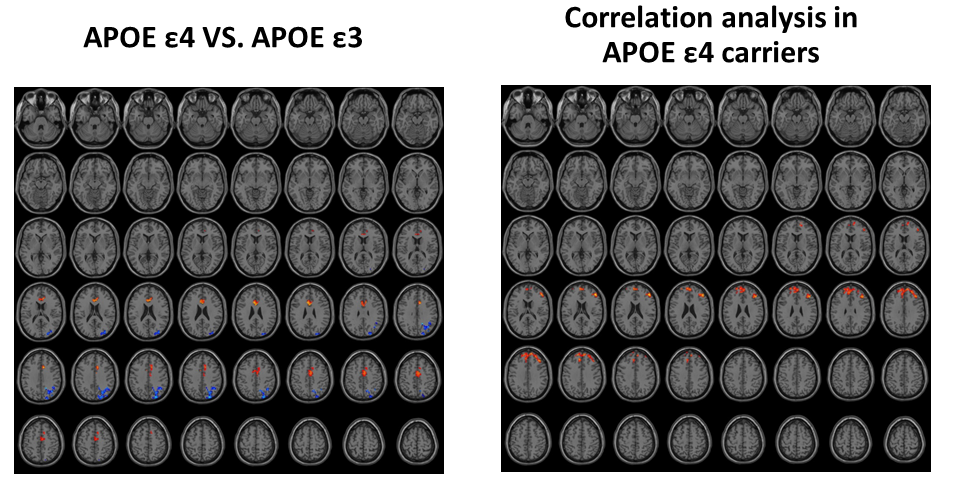


Abbreviation: APOE: apolipoprotein E; fALFF: fractional amplitude of low frequency fluctuations

Figure s7. The effect of APOE ε2 on fALFF


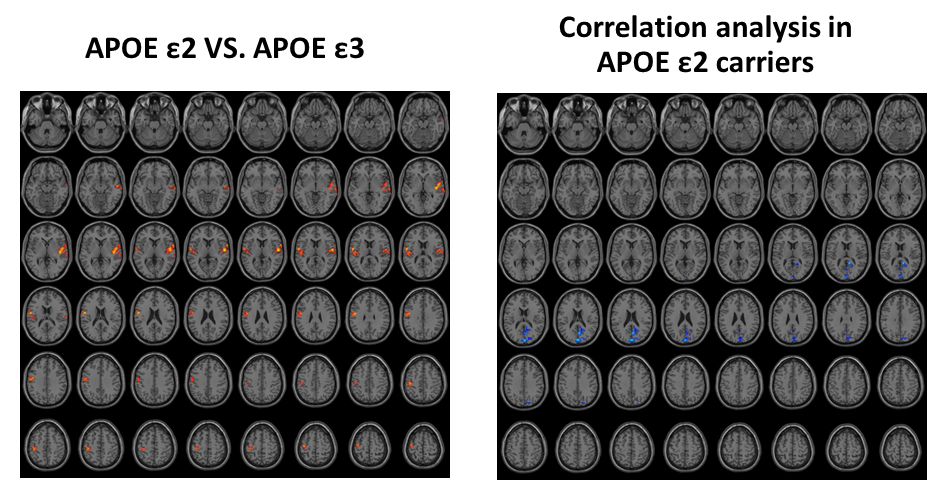


Abbreviation: APOE: apolipoprotein E; fALFF: fractional amplitude of low frequency fluctuations

As for GMV analysis, no significant differences were found. This partly suggests that gray matter volume is a relatively insensitive indicator.

These findings mostly locate within the DMN that are in line with previous studies on APOE and PHS. For example, one previous study found that PHS was associated with amyloid SUVR within frontal cortical regions and volume loss in the entorhinal cortex, inferior parietal cortex, inferior and middle temporal cortex, even after controlling for APOE status [9]. Studies focusing on APOE found that APOE ε4 was associated with brain atrophy, amyloid deposition, and functional connectivity decrease in the DMN [10, 11], while APOE ε2 carriers had a lower global Aβ burden [12].

In summary, the qualitative analysis showed the coexistence of both overlapped and different effects of PHS and APOE on three neuroimaging features. This is reasonable considering the followed two points: 1). The two indexes are somehow different. APOE is a single gene, while PHS covers 31 risk genes (including APOE ε2 and ε3). PHS involves multiple facets of the AD pathological process, making the functional changes more complex; 2). The method of supplementary analysis is a little different since APOE and PHS are two kinds of data.

**Supplementary Material 9.**

We supplementarily performed the fusion analysis in MCI and AD with positive amyloid (A+), as well as HC with negative amyloid (A-). Finally, we included 65 HC A-, 37 MCI A+, 18 AD A+ (Table s8). The overall results keep largely the same with original findings and help to support our original conclusions.

To be specific, MCCAR+ jICA was used to perform the 3-way (fALFF, GMV, and amyloid SUVR) fusion analysis. One joint component was identified that was correlated with PHS. The resulting spatial maps were Z-transformed and visualized at |Z|>2 in Figure s8. Along AD-continuum, PHS was correlated with 1) decreased fALFF in the IPL and frontal regions, while increased fALFF in the ACC and insula; 2) decreased GMV in the IPL, subcortical regions, and temporal region, while increased GMV in the insula, and occipital regions; 3) increased amyloid SUVR in the IPL, and frontal regions, while decreased amyloid SUVR in the subcortical regions. Furthermore, the loadings of ICs were positively correlated with PHS (r = 0.30, p = 0.001 for fALFF; r = 0.34, p < 0.001 for GMV; r = 0.52, p < 0.001 for amyloid SUVR). Significant differences in loadings of GMV and amyloid SUVR among groups were also observed. Conclusively, these results are largely consistent with our original findings and help to support our study.

Table s8. Demographic information

| **Demographic characteristics** | **HC A-** | **MCI A+** | **AD A+** | **F-value/X^2^** | **Sig** |
| --- | --- | --- | --- | --- | --- |
|  | **N=65** | **N=37** | **N=18** |  |  |
| Age | 76.66±5.97 | 78.73±7.67 | 80.45±7.31 | 2.67 | .073 |
| Gender (F/M) | 30/35 | 19/18 | 7/11 | 0.77 | 0.68 |
| Education | 16.85±2.36 | 15.46±2.41 | 15.33±2.87 | 5.05 | 0.008ab |
| GDS | 0.74±0.96 | 1.38±1.11 | 2.50±1.58 | 18.24 | <0.001abc |
| PHS | -0.30±0.29 | 0.46±0.88 | 0.58±0.63 | 28.27 | <0.001ab |
| Cognitive Scores | | | | | |
| MMSE | 29.14±1.11 | 27.70±1.85 | 18.61±5.46 | 130.28 | <0.001abc |
| CDR global | 0.00±0.00 | 0.50±0.00 | 1.19±0.57 | 226.85 | <0.001abc |
| CDR sum | 0.03±0.12 | 1.70±1.03 | 6.75±2.99 | 195.50 | <0.001abc |
| ADNI_MEM | 1.13±0.66 | 0.15±0.45 | -0.97±0.70 | 92.79 | <0.001abc |
| ADNI_EF | 1.15±0.86 | 0.32±0.89 | -1.06±0.99 | 41.93 | <0.001abc |
| ADNI_LAN | 1.00±0.64 | 0.19±0.83 | -0.91±1.35 | 39.75 | <0.001abc |
| ADNI_VS | 0.23±0.60 | -0.05±0.77 | -0.92±1.21 | 15.65 | <0.001bc |

Data are presented as means ± standard deviations.

Abbreviation: HC: healthy control; MCI: mild cognitive impairment; AD: Alzheimer's disease; APOE: apolipoprotein; GDS: geriatric depression scale; PHS: polygenic hazard score; MMSE, Mini-Mental State Examination; CDR: clinical dementia rating; ADNI-MEM: the composite scores for memory in ADNI; ADNI-EF: the composite scores for executive function in ADNI; ADNI-LAN: the composite scores for language in ADNI; ADNI-VS: the composite scores for visuospatial function in ADNI

a-c: post-hoc analysis further revealed the source of ANOVA difference (a: HC vs. MCI; b: HC vs. AD; c: MCI vs. AD) (p<0.05, significant difference between groups).

Figure s8. The identified joint component


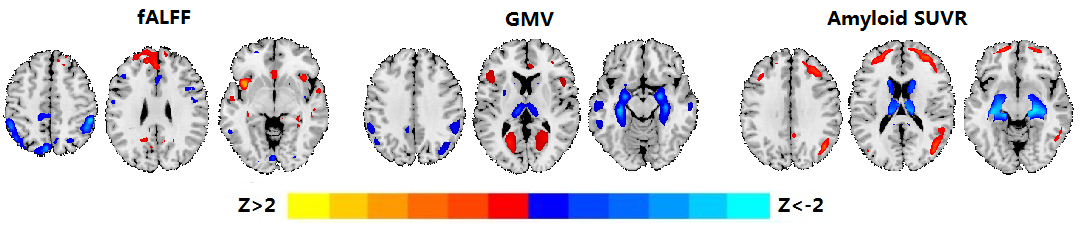


The spatial maps are visualized at |Z|>2, where the positive Z-values (red regions) mean higher fALFF, GMV, and more amyloid deposition, and negative Z-values (blue regions) indicate decreased fALFF, GMV, and less amyloid deposition.

Abbreviation: fALFF: fractional amplitude of low frequency fluctuations; GM: gray matter; SUVR: standard uptake value ratios

**Reference**

[1] Sheikh JI Yesavage JA (1986) Geriatric Depression Scale (GDS): recent evidence and development of a shorter version. *In: Brink TL, ed. Clinical Gerontology: A Guide to Assessment and Intervention. New York, NY: The Haworth Press;* , 165–173.

[2] Bondi MW Edmonds EC, Jak AJ, Clark LR, DelanoWood L, McDonald CR, Nation DA, Libon DJ, Au R, Galasko D, Salmon DP (2014) Neuropsychological criteria for mild cognitive impairment improves diagnostic precision, biomarker associations, and progression rates. *J Alzheimers Dis* **42**, 275-289.

[3] G. McKhann D. Drachman, M. Folstein, R. Katzman, D. Price, E.M. Stadlan (1984) Clinical diagnosis of Alzheimer's disease: report of the NINCDS-ADRDA Work Group under the auspices of Department of Health and Human Services Task Force on Alzheimer's Disease. *Neurology* **34**, 939-944.

[4] Crane P. K., Carle, A., Gibbons, L. E., Insel, P., Mackin, R. S., Gross, A., et al (2012) Development and assessment of a composite score for memory in the Alzheimer’s Disease Neuroimaging Initiative (ADNI). *Brain Imaging and Behavior*.

[5] Gibbons L. E., Carle, A. C., Mackin, R. S., Harvey, D., Mukherjee, S., Insel, P., et al (2012) A composite score for executive functioning, validated in Alzheimer’s Disease Neuroimaging Initiative (ADNI) participants with baseline mild cognitive impairment. *Brain Imaging and Behavior*.

[6] Reeve B. B., Hays R. D., Bjorner J. B., Cook K. F., Crane P. K., Teresi J. A., Thissen D., Revicki D. A., Weiss D. J., Hambleton R. K., Liu H., Gershon R., Reise S. P., Lai J. S., Cella D. (2007) Psychometric evaluation and calibration of health-related quality of life item banks: plans for the Patient-Reported Outcomes Measurement Information System (PROMIS). *Med Care* **45**, S22-31.

[7] Yu J. T., Tan L., Hardy J. (2014) Apolipoprotein E in Alzheimer's disease: an update. *Annu Rev Neurosci* **37**, 79-100.

[8] Leonenko G., Sims R., Shoai M., Frizzati A., Bossù P., Spalletta G., Fox N. C., Williams J., Hardy J., Escott-Price V. (2019) Polygenic risk and hazard scores for Alzheimer's disease prediction. *Ann Clin Transl Neurol* **6**, 456-465.

[9] Tan CH Bonham LW, Fan CC, et al (2019) Polygenic hazard score, amyloid deposition and Alzheimer's neurodegeneration. *Brain* **142**, 460-470. doi:410.1093/brain/awy1327.

[10] Li J. Q., Wang H. F., Zhu X. C., Sun F. R., Tan M. S., Tan C. C., Jiang T., Tan L., Yu J. T. (2017) GWAS-Linked Loci and Neuroimaging Measures in Alzheimer's Disease. *Mol Neurobiol* **54**, 146-153.

[11] Machulda M. M., Jones D. T., Vemuri P., McDade E., Avula R., Przybelski S., Boeve B. F., Knopman D. S., Petersen R. C., Jack C. R., Jr. (2011) Effect of APOE ε4 status on intrinsic network connectivity in cognitively normal elderly subjects. *Arch Neurol* **68**, 1131-1136.

[12] Salvadó G., Grothe M. J., Groot C., Moscoso A., Schöll M., Gispert J. D., Ossenkoppele R. (2021) Differential associations of APOE-ε2 and APOE-ε4 alleles with PET-measured amyloid-β and tau deposition in older individuals without dementia. *Eur J Nucl Med Mol Imaging*.
